# In Situ Mechanical Characterization of the Mixed-Mode Fracture Strength of the Cu/Si Interface for TSV Structures

**DOI:** 10.3390/mi10020086

**Published:** 2019-01-25

**Authors:** Chenglin Wu, Congjie Wei, Yanxiao Li

**Affiliations:** Department of Civil, Architectural, and Environmental Engineering, Missouri University of Science and Technology, Rolla, MO 65409, USA; cw6ck@mst.edu (C.W.); yl42y@mst.edu (Y.L.)

**Keywords:** TSV, nanoindentation, FIB, micro-cantilever beam, mixed-mode, fracture

## Abstract

In situ nanoindentation experiments have been widely adopted to characterize material behaviors of microelectronic devices. This work introduces the latest developments of nanoindentation experiments in the characterization of nonlinear material properties of 3D integrated microelectronic devices using the through-silicon via (TSV) technique. The elastic, plastic, and interfacial fracture behavior of the copper via and matrix via interface were characterized using small-scale specimens prepared with a focused ion beam (FIB) and nanoindentation experiments. A brittle interfacial fracture was found at the Cu/Si interface under mixed-mode loading with a phase angle ranging from 16.7° to 83.7°. The mixed-mode fracture strengths were extracted using the linear elastic fracture mechanics (LEFM) analysis and a fracture criterion was obtained by fitting the extracted data with the power-law function. The vectorial interfacial strength and toughness were found to be independent with the mode-mix.

## 1. Introduction

Thermal mechanical reliability plays a critical role in microelectronic devices, affecting their performance and service life spans. In situ mechanical characterizations are essential to predict the thermal–mechanical behaviors of these devices. The associated techniques and approaches rapidly emerge along the technology growth in 3D integrated circuits and devices [1,2,3,4,5,6,7]. One of the typical approaches is nanoindentation [6,8,9], which utilizes a small-scale probe with controlled force and displacement applied directly to the substrates or micro- and nanostructures [10,11]. Utilizing various sizes and shapes of the probe, the small-scale nonlinear material behavior can be characterized. This work focuses on the latest development of the nanoindentation techniques applied to 3D integrated microelectronic devices with a through-silicon via (TSV). 

As microelectronic devices become smaller and more complex, 3D integration becomes necessary for more efficient engineering and design. This integration consists of the micrometer copper vias passing through silicon die, serving as both electronical connections and mechanical supports. The copper vias are typically deposited by the electroplating approach and have complex grain structures. Under such conditions, the TSVs share different material properties, comparing to the bulk copper. Surface treatments are often conducted to the TSVs to avoid diffusion and enhance mechanical strength at Cu/Si interface. To have a comprehensive understanding of the mechanical behavior of the TSV and related interface, in situ small scale characterizations are required. 

Nanoindentations have been widely adopted for in situ characterization of mechanical properties of thin-films and nanostructured materials [6,7,8,9,10,11]. The elastic and plastic properties can be readily extracted using the force–displacement responses produced by nanoindentation with various tip shapes and sizes [12,13,14]. In addition, miniature specimens prepared using focused ion beam (FIB) fabrication techniques can also be utilized to obtain a more systematic understanding of the deformation mechanisms at small-scales. Therefore, the combination of nanoindentation and FIB fabrication presents a unique opportunity in probing the mechanical behavior of TSV structures and interfaces in 3D integrated microelectronic devices. In this paper, a cantilever beam approach for extracting the mixed-mode interface strength is proposed. Miniature cantilever beams with various lengths were fabricated using a FIB. Both analytical and numerical models were developed to extract the mixed-mode interfacial strength at the TSV/Si interface. The extracted results were then fitted with the power-law failure criterion [15,16,17,18] producing an input for failure prediction and reliability evaluations.

## 2. Materials and Sample Preparation

**Figure 1:**
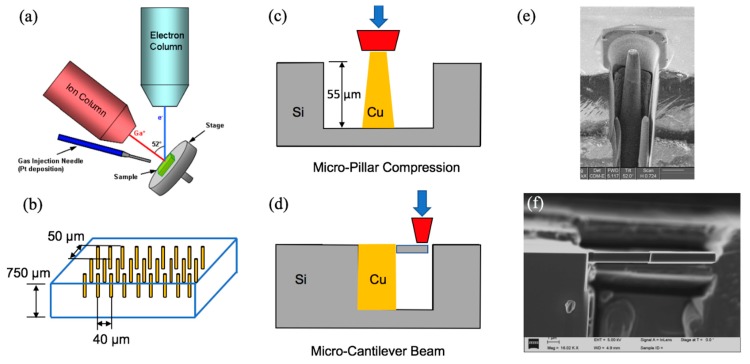
Through-silicon-via (TSV) specimens: (**a**) Focused ion beam scanning electron microscopy (FIB-SEM) dual beam system, (**b**) TSV in silicon substrate, schematics of (**c**) micro-pillar, (**d**) micro-cantilever experiments, SEM images of (**e**) micro-pillar adapted with permission from [8], (**f**) cantilever beam specimens.

**Figure 2:**
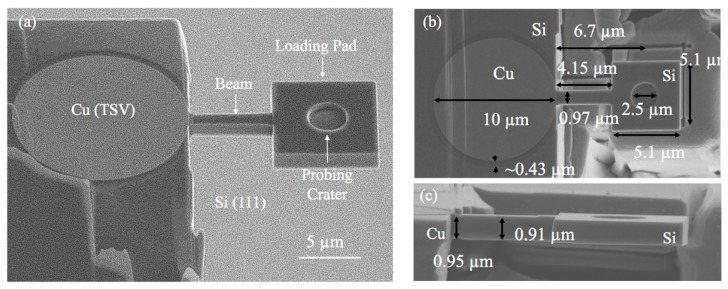
Micro-cantilever beam specimens: (**a**) Isometric view and structural components, (**b**) top view, and (**c**) side view with dimensional details (*L* = 4 µm).

The as-received TSV structure has periodic blind Cu arrays in a (001) Si wafer with a depth of 780 µm. The nominal via diameter and depth were 10 and 55 µm with a pitch spacing of 40 µm along the (110) direction and 50 µm along the (100) direction of the wafer, as illustrated in Figure 1. Two types of miniature specimens were prepared: The micro-pillar and cantilever beam specimens. The micro-pillar specimens were prepared by dicing and polishing the silicon wafer to have one row of the via away from the free surface by a distance of 20 µm. For each micro-pillar specimen, the top 100 nm was removed to avoid the effect of surface roughness. The silicon around the selected via was then subsequently removed, following a pattern of a concentric ring with a 3 µm thickness, as illustrated in Figure 1e. The inner ring was set at the same size as the via diameter, the outer ring was then about 16 µm in diameter. Due to the tapering effect, the top diameter of the via after the milling was about 6 µm, which formed 2 degrees of tapering angle along the via length. The micro-cantilever beam specimens were milled out of the silicon matrix near the copper via using a similar beam energy (ranging from 3–300 keV) used for the micro-pillar specimens. The side view of the prepared micro-cantilever beam is shown in Figure 1f. More details of the fabricated micro-cantilever beam are shown in Figure 2. A total of six types of micro-cantilever beam specimens were prepared with various lengths ranging from 1 to 30 µm. The width and height of the beam were set to be close to 1 µm. A specially designed square loading pad was also fabricated at the end of the beam with a size of 5.1 µm (note that the length of the loading pad was excluded from the total length to obtain the beam length). A probing crater with a diameter of 2.5 µm was carved into the loading pad to avoid the slipping of indenter tip during loading. At the Cu/Si interface, a pre-milled notch with a length of 100 nm was created, serving at the pre-crack. A total of 3 specimens were fabricated for each type of the micro-cantilever beams. 

## 3. Nanoindentation Experiment

The nanoindentation experiments were conducted using the Hysitron TI-95 Tribo-indenter® (Bruker Corporation, Billerica, MA, USA) on micro-cantilever specimens with a flat-punch tip having diameters of 2 µm. The micro-pillar results for analysis were obtained from our previous work [8]. The experimental details and subsequent extraction methods have been described in our previous work [8,19]. For the micro-cantilever beam experiment, the flat punch tip was placed inside the loading crater of the loading pad to apply displacement-controlled loading. A loading rate of 0.5 nm/s was applied until the contact between the cantilever beam and the sample’s surface was reached. It is worth noting here that the mechanical backlash was corrected during the tip-optic calibration process. A pre-loading with a maximum load of 1 µN was applied at the end of the beam to ensure proper contact.

## 4. Analysis

### 4.1. Plastic Behavior of Cu

**Figure 3:**
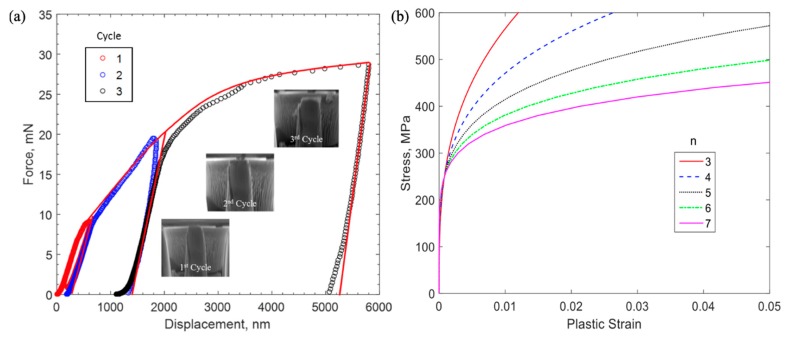
(**a**) Force–displacement response of micro-pillar experiment, reproduced with permission from [8], and (**b**) stress–plastic strain relationship from Ramberg-Osgood relationship.

The force–displacement response obtained from a previous experiment [8] is shown in Figure 3a. Observing these results from the previous work, significant plastic responses were observed, as indicated by the permanent deformation after each unloading. As explained in the previous work [8], the residual deformations were also confirmed by the SEM images, shown in Figure 3a. To extract this observed elastic–plastic property, a finite element analysis was conducted, considering the tapering caused by non-uniform stress distribution [8,19]. The Ramberg–Osgood power-law relationship [20] was adopted in the numerical models to compare with experimental results. The J-2 flow theory was used to model the Cu plasticity. The 4-node quadrilateral axis-symmetrical elements in commercial finite element code ABAQUS® (Abaqus Inc., Providence, RI, USA) were used for the finite element modeling. The Ramberg–Osgood power-law relationship has been widely used for the description of plastic strain hardening of nanoindentation experiments, the stress versus plastic strain curve based on this law showed good agreement with experimental data [21,22,23]. In this relationship, the stress versus plastic strain response follows the description below,

(1) $$\varepsilon_{p}=\;\frac{3}{7}\frac{\sigma_{e}}{E}{\left(\frac{\sigma_{e}}{\sigma_{0}}\right)}^{n-1}$$

where $\varepsilon_{p}$ is the plastic strain, $\sigma_{e}$ is the equivalent stress, $\sigma_{0}$ is the yield stress (which is found to be around 216 MPa), *n* is the Ramberg–Osgood parameter—which was found to be three from the fitting results [8,19]—and *E* is Young’s modulus (which is found to be 110 GPa), were obtained with the Oliver–Pharr approach, using a conical probe has a tip radius of 500 nm. This method is well applied to axis-symmetrical indenter geometries. The reduced modulus is given by:

(2) $$E_{r}=\frac{\sqrt{\pi }}{2\sqrt{A\left(h_{c}\right)}}S$$

where, $S={\left(\frac{dP}{dh}\right)}_{P_{max}}$ is the contact stiffness obtained from test data, $A\left(h_{c}\right)$ is the contact area at contact depth, $h_{c}$, given by $h_{c}=h_{max}-\frac{\varepsilon P_{max}}{S}$. ε equals 1 for flat-ended punch. The Young’s modulus can then be obtained with:

(3) $$E=\frac{1-v^{2}}{\frac{1}{E_{r}}-\frac{1-v_{i}^{2}}{E_{i}}}$$

where v and $v_{i}$ are the Poisson ratio of sample and indenter, respectively. 

**Figure 4:**
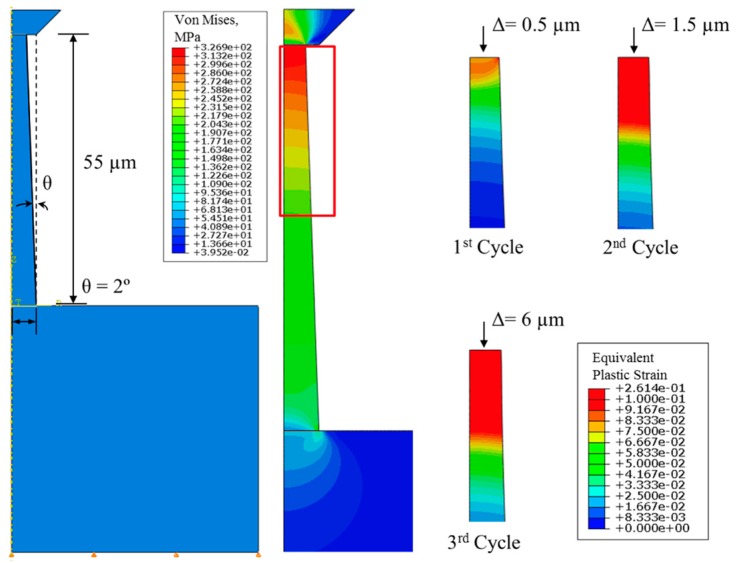
Finite element analysis of micro-pillar experiment, reproduced with permission from [8].

The extracted elastic–plastic properties of the copper were used to evaluate the fracture strength at the Cu/Si interface. More analysis details are shown in Figure 4, where both the von Mises stress and equivalent plastic strains show non-uniform distributions. The non-uniform distribution was an indication of the tapering effect and further demonstrated the needs of conducting finite element analysis (FEA) to extract the plastic properties of Cu. This result also shows that the nonlinear force–displacement response has geometrical effects. Stress and plastic strain contours for FE modeling of micro-pillar compression are shown in Figure 4, where yield stress and the Ramberg–Osgood parameter are taken to be 216 MPa and 3, respectively. The slight gradient shown in the contour plots was due to the tapered cross-section of the specimen.

### 4.2. Micro-Cantilever Experiment

#### 4.2.1. Failure Surface Characterization

**Figure 5:**
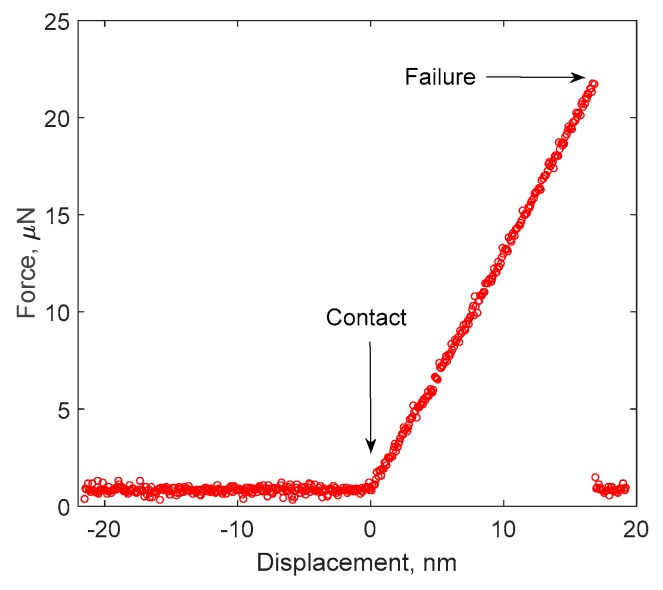
Typical force-displacement response of micro-cantilever beam (*L* = 4 µm).

**Figure 6:**
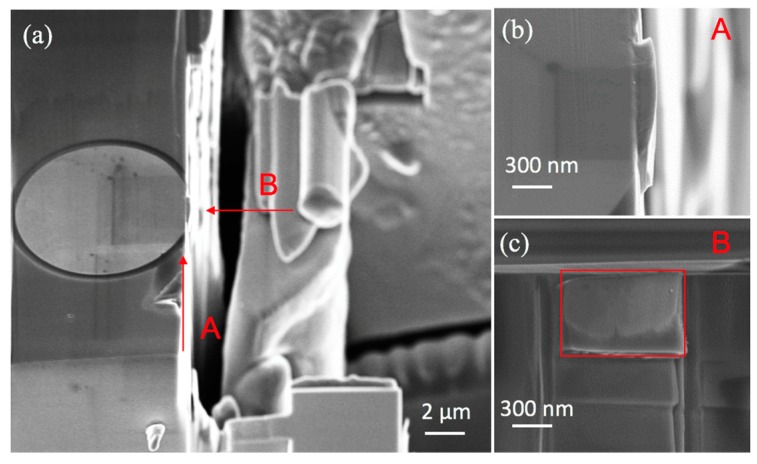
(**a**) SEM images of post-failure of cantilever beam, (**b**) top view details, (**c**) interfacial details (element analysis conducted within the red-boxed region).

The force versus displacement response for a typical micro-cantilever beam specimen is shown in Figure 5. The early contact was established as shown by the turning point between the approaching and loading response. A linear response was observed followed by a sudden failure, in terms of the drop of the force from the peak value to zero. This sudden force drops indicated a brittle Cu/Si interface. The failed surface shown in Figure 6 was characterized using SEM and energy dispersive spectroscopy (EDS) as labeled out with the red box. The elements and weight percentage results are shown in Table 1. As listed, most of the elements detected were Cu, which was followed by Si and elements in the liner materials at the TSV/Cu interface (Fe, Ta, Os). This result is similar to that of the shear failure surface from the previous work [8]. As previously concluded, the majority of the Cu signal comes from the background Cu materials in the TSV, which indicated an interfacial failure locus within the silicon matrix. The Young’s modulus for Cu and Si are 110 and 165 GPa, respectively. The shear modulus used in the analysis for Cu and Si are 42.3 and 64.45 GPa, respectively, the Poisson’s ratios are 0.3 and 0.28, respectively.

#### 4.2.2. Mixed-Mode Fracture

(1) Linear Elastic Fracture Mechanics (LEFM) Analysis

The stress analysis for the micro-cantilever beam experiment was conducted using both LEFM and the non-linear fracture mechanical model (NLEFM), considering the effect of Cu plasticity. Analytically, the far-field load-generated near-field stress had both normal ($\sigma_{0}$) and shear ($\tau_{0}$) components. From the Euler beam theory and ignoring the nonlinear shear deformation caused by root rotation, these stresses can be obtained using the beam geometry and material constants of the silicon.

(4) $$\sigma_{0}=\frac{PL}{6bh^{2}},\;\tau_{0}=\frac{3P}{2bh}$$

The local stress at the crack-tip can then be computed using the near-field stress and the stress intensity factor as

(5) $$K=K_{I}+iK_{II}$$

(6) $$\sigma =\frac{Re\left(Ka^{i\epsilon }\right)}{\sqrt{2\pi l}},\;\tau =\frac{Im\left(Ka^{i\epsilon }\right)}{\sqrt{2\pi l}}$$

where a is the crack length, $\epsilon =\frac{1}{2\pi }ln\left(\frac{1-\beta }{1+\beta }\right)$, $\beta =\frac{1}{2}\frac{\mu_{1}\left(1-2v_{2}\right)-\mu_{2}\left(1-2v_{1}\right)}{\mu_{1}\left(1-v_{2}\right)+\mu_{2}\left(1-v_{1}\right)}$ are the materials mismatch parameters [24,25], $\mu_{i},\;v_{i}$ are the shear modulus and Poisson’s ratio for Cu and Si, respectively, where i=1, 2, 1 represents Cu, 2 represents Si. l=100 nm is the length scale for the investigated problem. The stress intensity factors were obtained using the LEFM FEA analysis. 

**Figure 7:**
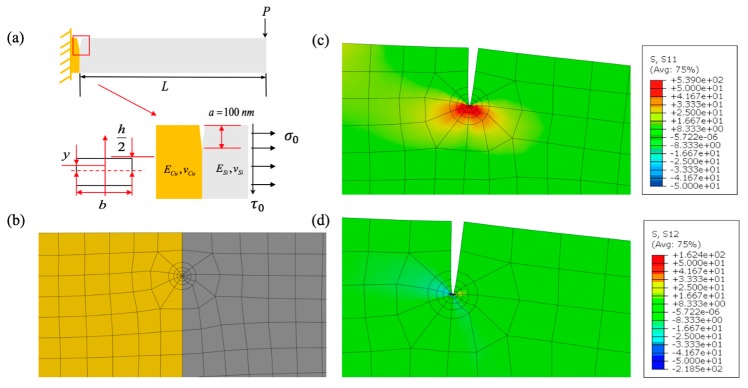
(**a**) Illustration of micro-cantilever beam with pre-crack, (**b**) FEA mesh details (yellow indicates Cu, grey indicates Si), (**c**) normal stress at the crack tip, (**d**) shear stress at the crack tip.

The mesh details for the LEFM finite element analysis are shown in Figure 7a, where the plain strain 4-node bilinear quadrilateral elements were used in the region away from the crack-tip. The size of FE meshes was chosen to be less than 1/3 of pre-notch length, which was set as 100 nanometers. The mesh configuration used in this mode provided four contour integral paths to calculate J-integrals. The singular elements were then used near the crack-tip with a square root singularity [26]. The normal, shear stress and strain contours of the analyzed micro-cantilever beams are shown in Figure 7b. The analysis was then conducted for the six types of specimens with given tested failure loads (P) and geometrical characteristics. The phase angle was defined in terms of stress [25] as $\psi =\arctan\left(\frac{Im\left(Ka^{i\epsilon }\right)}{Re\left(Ka^{i\epsilon }\right)}\right)$ and plotted against the thickness-over-length ratio for the cantilever beams. The results (Table 2) showed that the variation in the beam height-over-length ratio provides a phase angle ranging from 16.7° to 83.7°, covering almost the half range of the mode-mix, ranging from 0 to +90 degrees. The normal and shear stress ($\sigma_{0},\;\tau_{0}$) obtained using Equation (6) at the failure load are then the mixed-mode fracture strength corresponding with the associated phase angle. The vectorial fracture strength can also be obtained by $Τ=\sqrt{\sigma_{0}^{2}+\tau_{0}^{2}}$. The fracture toughness was also calculated using the critical stress intensity factors calculated following the equation [24] below,

(7) $$\Gamma =\frac{\left(1-\beta^{2}\right)}{E_{*}}\left(K_{Ic}^{2}+K_{IIc}^{2}\right)$$

It should be noted here that these crack-tip stresses are essentially the stresses at l away from the crack-tip. The effect of the plastic zone was omitted, since the calculated stresses at these distances were much smaller than the yield strength of the Cu (216 MPa). However, the NLEFM analysis was nevertheless conducted to justify the negligence of the plastic effect.

(2) NLEFM Analysis

**Figure 8:**
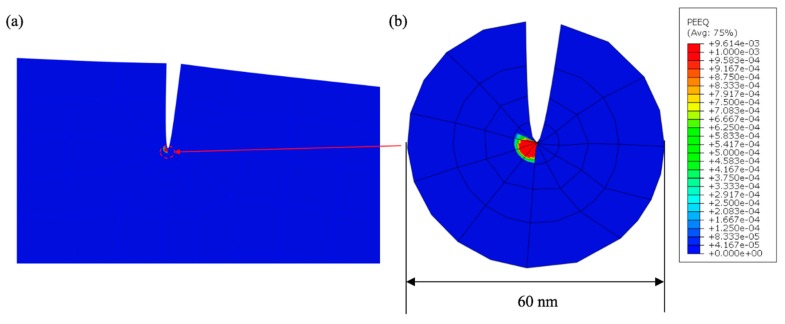
Non-linear fracture mechanical mode (NLEFM) analysis results of micro-cantilever beam for *L* = 4 µm: (**a**) Far-field view, (**b**) localized view near crack-tip showing equivalent plastic strain contour.

The non-linearity of the interfacial mixed-mode fracture typically comes from two perspectives: The cohesive behavior at the interface and the material’s non-linearity. Based on our previous work, we concluded that the cohesive zone for the investigated Cu/Si interface was smaller than 100 nm. Therefore, the cohesive zone analysis was not considered, since the cohesive zone length was much smaller than the characteristic length of the micro-cantilever beam. However, the material nonlinearity, in this case the Cu plasticity, had to be considered in the modeling to ensure the results obtained using LEFM were valid. In the NLEFM analysis, same geometrical characteristics and mesh configuration were used as in the LEFM. The only modification was the replacement of the elastic behavior of Cu with the measured elastic–plastic behavior from the micro-pillar experiment. All six types of specimens were modeled by applying the measured failure loads. The typical equivalent plastic strain contours are presented in Figure 8. The region where material has entered the plastic regime is labeled by the red dashed circles. The radius of these circles ranged from 10 to 15 nm, which were smaller than the 100 nm characteristic length scale used in the LEFM analysis, which validated the obtained mixed-mode fracture results. 

## 5. Results and Discussions

### 5.1. Strain Hardening of Cu Via

The yield strength measured from the micro-pillar experiment was close to those measured at the bulk scale. However, the Ramberg–Osgood parameter (*n* = 3) measured at micro-scale was much less than those typically measured at bulk scale (*n* = 5), which indicated a possible size effect caused by the reduced relative grain size. The average grain sizes measured for the TSV used in this study was about 500 nm [8], which was slightly smaller than the typical grain size observed at the bulk scale. The smaller grain size increased the total grain boundary area that contributed to the strain hardening mechanism, as illustrated by Taylor’s theory [27,28,29,30,31]. This increased strain hardening behavior of Cu can effectively “lock” the plastic strain development within a small region, as observed in shear fracture of our previous work as well as in the micro-cantilever beam experiment. Therefore, it is worth noting here that the Cu plasticity had limited effects on the interfacial fracture of Cu/Si interface. The NLEFM results also confirmed that the crack-tip induced stress singularity caused a limited plastic effect. This however, was constrained within an area smaller than the characteristic length of the investigate interface. This constrain was also related to the limitation on the mode-mix, induced by varying the length-over-height ratio of the micro-cantilever beam. The pure mode-I and mode-II cases were not fully achieved, though closely approximated, avoided the growth of the plastic zone in the Cu via.

### 5.2. Mixed-Mode Cu/Si Interfacial Behavior

**Figure 9:**
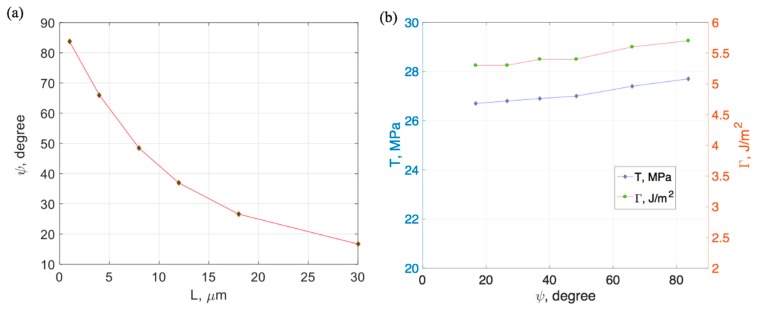
(**a**) Phase angle versus beam length, (**b**) vectorial fracture strength and toughness versus phase angle.

The phase angle versus the beam length (*L*) is plotted in Figure 9a. A decreasing trend was observed as the beam length increased. The range of the phase angle was from 16.7 to 83.7 degrees, covering most parts of the positive mode-mix (0–90°), which indicated completeness of the experimental data set in determining the fracture criterion at the Cu/Si interface. 

The mode-mix (in terms of phase angle) versus the vectorial interfacial strength (*T*) and the interfacial toughness (Γ) is shown in Figure 9b. A slight increase was observed for both values as the phase angle increased (i.e., more shear contribution is present). However, both the strength and toughness are relative, independent of the mode-mix. The average vectorial mixed-mode strength was found to be less dependent on the mode-mix. The average failure strength (|T|) was about 27 MPa, which was much lower than the yield strength of Cu (216 MPa) and the fracture strength of Si. Therefore, we suspected the liner materials at the Cu/Si interface contributed to this low interfacial strength. 

**Figure 10:**
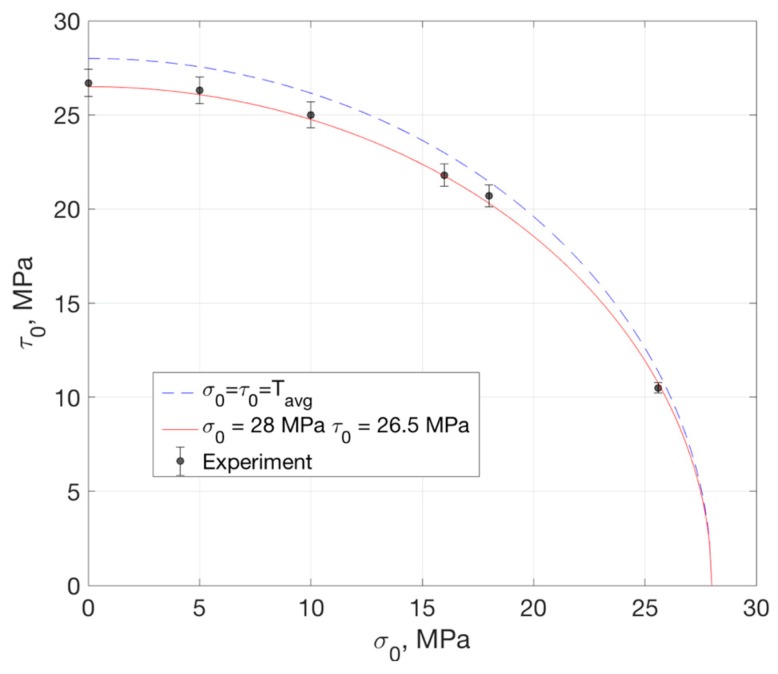
Failure criterion and experimental data.

Given these results, a fracture criterion was then proposed for the tested Cu/Si interface. Following the power-law failure criterion proposed by Carlsson et al. [15], the failure strength of the Cu/Si interface can be described as the following equation.

(8) $${\left(\frac{\sigma_{0}}{\sigma_{c}}\right)}^{\lambda }+{\left(\frac{\tau_{0}}{\tau_{c}}\right)}^{\lambda }=1$$

where $\sigma_{c},\;\tau_{c}$ are the fracture strengths for pure mode-I (normal) and mode-II (shear) and λ is a fitting parameter, which was set at 1.8. The measured experimental data are then fitted with the proposed failure criterion, as shown in Figure 10. The dashed blue line shows the fitting of experimental data with $\sigma_{c}=\tau_{c}=T_{avg}$. The red solid line shows the fitting of $\sigma_{c}=28\;\mathrm{MPa},\;\tau_{c}=26.5\;\mathrm{MPa}$. The better fitting of the experimental was observed when setting different fracture strengths for pure mode-I and mode-II. These results indicated that the mode-mix still had a moderate effect on the fracture strength, although the vectorial value stayed almost constant. When the combined normal and shear stress in the plane satisfy the condition of ${\left(\frac{\sigma }{\sigma_{c}}\right)}^{1.8}+{\left(\frac{\tau }{\tau_{c}}\right)}^{1.8}<1$, the fracture initiation was not likely to occur.

## 6. Conclusions

This work combines the micro-pillar compression and micro-cantilever experiments to extract the mixed-mode fracture strength of the Cu/Si interface at the small scale (<100 nm). A series of micro-cantilever beam specimens with various beam lengths were fabricated and tested covering almost the full range of the positive mode-mix (0–90°). The following conclusions were drawn from the experiment and analysis results:

(1) The mixed-mode fracture at the Cu/Si interface is brittle in nature. No significant cohesive zone was observed, nor does the plastic hinge that is typically found for the micro-cantilever beam consist of metal materials.
(2) The effect of the plastic behavior of the Cu was negligible. The NLEFM analysis results showed that this is mostly due to the constrained plastic region, which was much smaller than the characteristic length (100 nm).
(3) The vectorial fracture strength and toughness were obtained from the analysis, which indicated that the effect of the mode-mix was limited, which was suspected to be related to the constrained plastic zone.
(4) A power-law fracture strength criterion could be used to fit the experimental data with close agreement. The criterion could be used for the design and engineering of TSV structures in combined loadings.

Future studies will focus on the evaluation of the effects of residual stress, interfacial defects, and impurities of Cu on the mixed-mode interfacial strengths. In particular, the residual stress built-up at the Cu/Si interface, due to elastic and coefficient of thermal expansion (CTE) mismatch, will cause deviations on the extracted mixed-mode fracture strengths measured. Under extreme circumstances, plastic zones can be formed at the interface, as well providing the possible reduction in the mixed-mode fracture strengths. The residual stress generated during the fabrication process will be estimated and taken into consideration in future studies.

## Figures and Tables

**Table 1:** Element and weight percentage of energy dispersive spectroscopy (EDS).

Element	Weight %
Si	5.62
Fe	0.02
Cu	84.34
Ta	1.04
Os	0.01

**Table 2:** Mixed-mode fracture analysis results.

*L* (µm)	ψ (Degree)	$\sigma_{0}$ (MPa)	$\tau_{0}$ (MPa)	*T* (MPa)	Γ (J/m^2^)
1	83.7	10.5	25.6	27.7	5.7
4	66.0	20.7	18.0	27.4	5.6
8	48.4	21.8	16.0	27.0	5.4
12	36.9	25.0	10.0	26.9	5.4
18	26.6	26.3	5.0	26.8	5.3
30	16.7	26.7	0.0	26.7	5.3
